# Effect of the Polypill on Adherence and Prevention of Cardiovascular Diseases in Patients With or at High Risk of Cardiovascular Diseases: A Meta-Analysis of Randomized Controlled Trials

**DOI:** 10.7759/cureus.34134

**Published:** 2023-01-24

**Authors:** Rahat A Memon, Bansari Raveena Bai, FNU Simran, Meena Kumari, FNU Aisha, Kondabolu Sai Kiran, Yasitha Kakarlapudi, Faraz Saleem

**Affiliations:** 1 Internal Medicine, Abington Memorial Hospital, Abington, USA; 2 Internal Medicine, People's University of Medical & Health Science for Women, Nawabshah, PAK; 3 Neurology, Internal Medicine, Ghulam Muhammad Mahar Medical College, Sukkur, PAK; 4 Internal Medicine, Dow University of Health Sciences, Karachi, PAK; 5 Medical College, Liaquat University of Medical and Health Sciences, Hyderabad, PAK; 6 Internal Medicine, Andhra Medical College, Visakhapatnam, IND; 7 Internal Medicine, Akhtar Saeed Medical and Dental College, Lahore, PAK

**Keywords:** meta-analysis, adherence, fixed dose combination, cardiovascular diseases, polypill

## Abstract

It is important to understand the role of polypills on medication adherence and clinical outcomes in patients with or at high risk of cardiovascular diseases (CVD) to help decision-makers make robust guidelines on using polypills in these people. Therefore, the current meta-analysis was conducted to assess the impact of polypills on medication adherence and clinical outcomes in patients with or at high risk of CVD. The present meta-analysis was performed according to the Preferred Reporting Items for Systematic Reviews and Meta-analyses (PRISMA) guidelines. We searched the electronic databases PubMed, Embase, and the Cochrane Library for randomized control trials (RCTs) from inception to January 1, 2023. The outcomes assessed in the present meta-analysis were adherence to prescribed drugs at the study end, the incidence of cardiovascular events, and change in low-density lipoprotein cholesterol (LDL-C), total cholesterol, and high-density lipoprotein (HDL) from baseline to the study end in mmol/l. A total of six studies were included in the present meta-analysis, with a total sample size of 13139 (6577 in the polypill group and 6562 in the control group). Meta-analysis showed that medication adherence was significantly higher in patients receiving polypills compared to the control group (relative risk (RR): 1.38, 95% confidence interval (CI): 1.22-1.56, p-value: 0.001). The risk of a cardiovascular event was significantly lower in the polypill group (RR: 0.72, 95% CI: 0.63-0.82, p-value: 0.001). No significant differences were found in the changes in LDL-C and total cholesterol between the two groups. This meta-analysis shows a significant impact of polypills on medication adherence. We also found that polypills can reduce the incidence of cardiovascular events in patients with or at high risk of CVD. Our study has also shown that regardless of the history of CVD, polypills play an important role in promoting medication adherence in patients with and without a history of CVD.

## Introduction and background

Poor medication adherence is one of the most common problems among patients with chronic diseases [1]. Many patients discontinue their medication before the intended end of the therapy, and just half of the prescribed dosages are consumed [2]. Different factors can lead to poor medication adherence, like poor communication between the physician and the patient or patients perceiving treatment as redundant. Patients could also believe that the advantages of their medication do not outweigh the side effects, or they might forget [3]. This leads to complications, extra healthcare costs, therapeutic failure, and adverse effects. Thus, improving medication adherence is an important factor in enhancing the chances of positive therapeutic outcomes.

Mortality from cardiovascular diseases (CVD) is still increasing in developing countries [4]. In developed countries, mortality rates due to CVD are stable or even decreasing because of the appropriate administration of drug treatments like antithrombotic agents, antihypertensive medications, and statins in patients at high risk of developing CVD [5]. According to estimates, the proper use of cardiovascular (CV) drugs for secondary prevention may have contributed to half of the overall decline in CVD mortality in developed nations over the past 20 years [6]. Lack of medication adherence is one of the fundamental factors that affects the strategies for CVD prevention. It has been estimated that adherence to cardiovascular medications is about 57% after a median of two years [7].

Patients with chronic diseases often take multiple pills every day for a long time, resulting in poor adherence to their drugs [8]. This happens particularly with cardiovascular diseases (CVD), when patients are more likely to forget to take their medications because they do not immediately experience the symptoms of their illness [9]. To enhance medication adherence, a strategy based on a fixed-dose combination, also known as a "polypill," includes important medications to reduce the risk of cardiovascular disease as a once-daily dose pill [10]. The polypill is a technological innovation that can improve medication adherence by simplifying pharmacotherapy [2]. Multiple medications are combined into one formulation instead of being taken as two or more pills separately [11-12].

It is important to understand the role of polypills on medication adherence and clinical outcomes in patients with or at high risk of CVD to help decision-makers make robust guidelines on using polypills for these people. Therefore, the current meta-analysis was conducted to assess the impact of polypills on medication adherence and clinical outcomes in patients with or at high risk of CVD.

## Review

Methodology

The present meta-analysis was performed according to the Preferred Reporting Items for Systematic Reviews and Meta-analyses (PRISMA) guidelines.

Data Sources and Searches

We searched the electronic databases PubMed, Embase, and the Cochrane Library for randomized control trials (RCTs) from inception to January 1, 2023, using terms including "polypill OR fixed-dose combination" AND "cardiovascular disease" AND "medication adherence". In addition, reference lists of all eligible studies were manually searched. Two investigators independently searched the databases to find relevant articles. Any disagreement between the two investigators was resolved via discussion.

Inclusion and Exclusion Criteria

We included RCTs that assessed the impact of polypills or fixed-dose combination strategies on medication adherence in patients with or at high risk of CVD. In our meta-analysis, the comparison group or control was usual care, in which drugs were prescribed separately. No restrictions were placed on the year of publication. All articles required a comparison between the high- and low-pill burden groups. It means that one group had to take more pills compared to the other group. It was also necessary that the studies deal with solid dosage formulations rather than any other dosage form. We excluded observational studies, quasi-experimental studies, and case reports. We also excluded studies with a follow-up period of fewer than six months.

Study Selection and Data Extraction

Studies identified through online databases were incorporated into a single EndNote X7 file. After removing duplicates, the articles were screened using the abstracts and titles by two independent authors. The full text of the eligible records was retrieved and assessed for the pre-specified inclusion and exclusion criteria. A risk of bias assessment was performed for all selected articles using the Cochrane risk-of-bias tool for randomized trials, version 2, by two investigators. We extracted data from all selected articles using data extraction developed in Microsoft Excel. We obtained study characteristics, including author name, year of publication, groups, sample size, follow-up duration, outcomes, and participants’ characteristics (age and gender). Any disagreement between the two authors in the process of study selection, risk of bias assessment, and data extraction were resolved via discussion or by a third author.

Outcome Measures and Quality Assessment

The outcomes assessed in the present meta-analysis were adherence (as reported in individual studies) at the study end, the incidence of cardiovascular events, and change in low-density lipoprotein cholesterol (LDL-C), total cholesterol, and high-density lipoprotein (HDL) from baseline to the end of the study in mmol/L.

Quality assessments of all included studies were performed by two investigators independently using the Cochrane risk-of-bias tool. Domains were random sequence generation, allocation concealment, blinding of outcome assessment, blinding of personnel and participants, incomplete outcome data, selective reporting, and other possible biases. Each domain was categorized into those with a low, high, or unclear risk of bias. Any disagreement between the two investigators was resolved through discussion.

Statistical Analysis

Data extracted from the selected studies were noted into a Microsoft Excel sheet and later put into Review Manager Version 5.4 (The Nordic Cochrane Center, The Cochrane Collaboration Copenhagen, Denmark). We used a random effect model in our meta-analysis. We used a random effect or fixed effect model using the Mantel-Haenszel method of calculating the risk ratio (RR) along with a 95% confidence interval (CI) for all dichotomous outcomes. For continuous variables, we calculated the mean difference (MD) with a 95% confidence interval (CI). A p-value of <0.05 was considered to be statistically significant. We assessed between-study heterogeneity by computing the I-square statistics. I-square statistics of 25% or less were regarded as low heterogeneity, while I-square statistics of more than 75% were categorized as high heterogeneity. Cocharn-Q statistics was used to test for heterogeneity, and a p-value less than 0.1 was considered significant for heterogeneity.

Results

**Figure 1:**
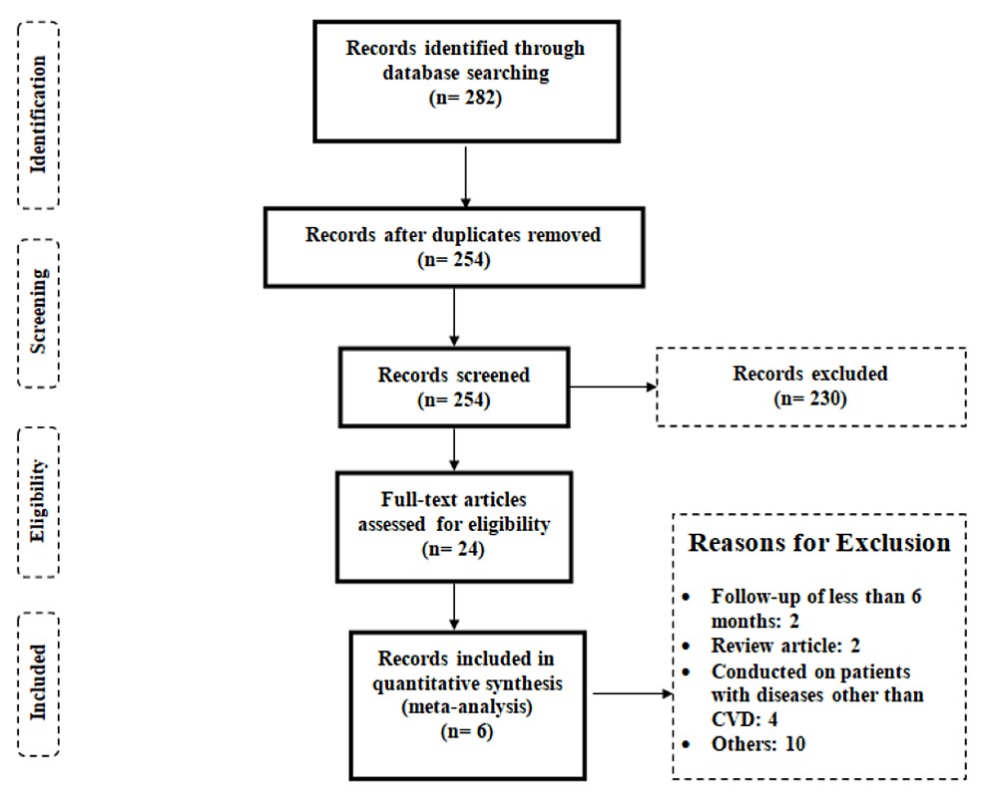
A PRISMA flowchart of the selection of studies

**Figure 2:**
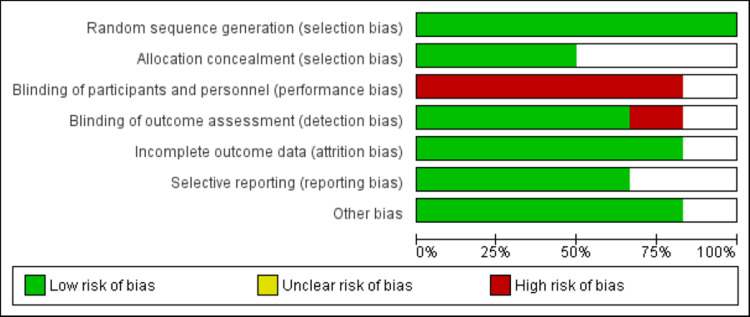
Risk of bias assessment

A flowchart of study selection is shown in Figure 1. The initial database search yielded 282 studies. After removing duplicates, 254 records were passed through initial screening using their titles and abstracts. Of them, 230 articles were excluded. Subsequently, 24 full-text articles were assessed based on pre-established eligibility criteria. As a result, a total of six studies were included in the present meta-analysis. Figure 2 shows the risk of bias assessment for all included studies. Overall, the risk of bias was moderate, as blinding participants was not possible in the included RCTs. However, out of the six included studies, the outcome assessor was blinded to the group to which patients were randomized in four studies.

Table 1 shows the characteristics of all the included studies. The pooled sample size was 13139 (6577 in the polypill group and 6562 in the control group). The follow-up of all included studies ranged from nine months to 60 months. Two RCTs only included patients with a history of CVD [10, 13], while four studies included patients who had a history of CVD as well as those who had a high risk of CVD [14-17]. 

**Table 1:** Study characteristics MI: myocardial infarction; CVD: cardiovascular disease

Author name	Year	Study population	Groups	Sample size	Medicine	Follow-up	Mean age (in years)	Male (%)	History of CVD (%)
Castellano et al. [10]	2014	Patients with a history of acute MI within the last two years.	Polypill	350	Aspirin (100 mg), ramipril (2.5, 5, or 10 mg), and atorvastatin (40 mg).	Nine Months	NR	NR	100%
			Control	345					
Castellano et al. [13]	2022	Patients with a history of acute MI within the last six months.	Polypill	1237	Aspirin (100 mg), ramipril (2.5, 5, or 10 mg), and atorvastatin (20 or 40 mg).	24 Months	NR	NR	100%
			Control	1229					
Patel et al. [14]	2015	People with or at high risk of CVD	Polypill	311	Aspirin (75 mg), simvastatin (40 mg), lisinopril (10 mg), and atenolol (50 mg).	12 Months	63.5	62.9%	61.1%
			Control	312					
Roshandel et al. [15]	2019	People with or at high risk of CVD	Polypill	3421	Hydrochlorothiazide (12·5 mg), aspirin (81 mg), atorvastatin (20 mg), and enalapril (5 mg).	60 Months	59.5	49.7%	10.8%
			Control	3417					
Selak et al. [16]	2014	People with or at high risk of CVD	Polypill	256	Aspirin (75 mg), simvastatin (40 mg), and lisinopril (10 mg), with either atenolol (50 mg) or hydrochlorothiazide (12.5 mg).	12 Months	62	63.5%	45.4%
			Control	257					
Thom et al. [17]	2013	People with or at high risk of CVD	Polypill	1002	Aspirin (75 mg), simvastatin (40 mg), and lisinopril (10 mg), with either atenolol (50 mg) or hydrochlorothiazide (12.5 mg).	13 Months	61.8	81.90%	76.2%
			Control	1002					

Medication Adherence

**Figure 3:**
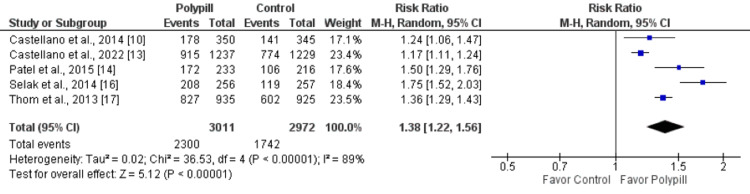
A forest plot showing the effect of the polypill on medication adherence Sources: References [10, 13-14, 16-17]

The primary outcome of the meta-analysis was medication adherence in patients with CVD or at high risk of CVD. Five studies addressed this outcome. In the polypill group, 76.5% of patients adhered to prescribed medications compared to 58.6% of patients in the control group. Medication adherence was significantly higher in patients in the polypill group (RR: 1.38, 95% CI: 1.22-1.56, p-value: 0.001), as shown in Figure 3. High heterogeneity was reported among the study results (I-square: 89%). High heterogeneity might be due to differences in the study population, as this meta-analysis included patients with and without a history of CVD. We performed a subgroup analysis to see the impact of polypills separately on patients with a history of CVD and patients without a history of CVD. Subgroup analysis has shown that adherence is significantly higher in the polypill group in patients with a history of CVD (RR: 1.26, 95% CI: 1.17-1.37) than in patients without a history of CVD (RR: 1.78, 95% CI: 1.45-2.19).

Cardiovascular Events

**Figure 4:**
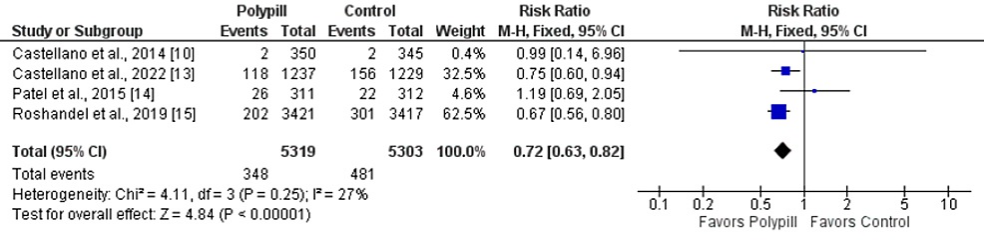
A forest plot showing the impact of the polypill on cardiovascular events Sources: References [10, 13-15]

Four studies compared the incidence of cardiovascular events among patients randomized into the polypill and control groups. The incidence of cardiovascular events was significantly lower in patients receiving polypills compared to the control group (RR: 0.72, 95% CI: 0.63-0.82, p-value: 0.001), as shown in Figure 4. No significant heterogeneity was reported among the study results (I-square: 27%, p-value: 0.25)

Total Cholesterol, LDL-C, and HDL

**Figure 5:**
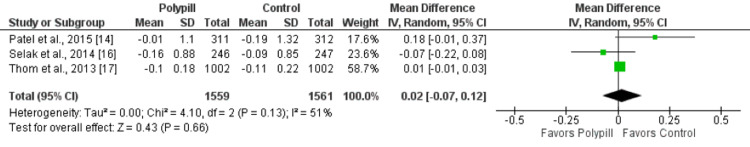
A forest plot showing the impact of the polypill on the change in total cholesterol Sources: References [14, 16-17]

**Figure 6:**
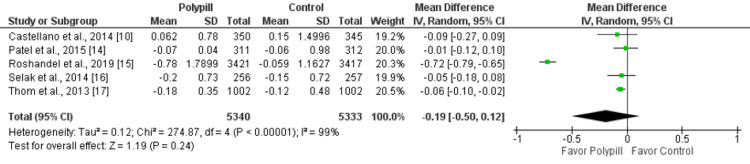
A forest plot showing the impact of the polypill on changes in LDL-C Sources: References [10, 14-17]

**Figure 7:**
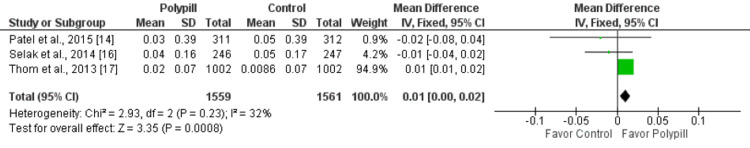
A forest plot showing the impact of the polypill on the change in HDL Sources: References [14, 16-17]

Meta-analysis of the studies of the effect of polypills on change in total cholesterol, which included 3110 patients, demonstrated no significant impact of polypills on change in total cholesterol (MD: 0.02, 95% CI: -0.007-0.12, p-value: 0.66) as shown in Figure 5. Similarly, no significant difference was reported between the two groups in terms of change in l LDL-C (MD: -0.19, 95% CI: -0.50-0.12, p-value: 0.24), as shown in Figure 6. We also assessed the change in HDL from baseline between the two groups, and the increase in HDL was significantly greater in patients receiving polypill compared to their counterparts (MD: 0.01, 95% CI: 0.00-0.02, p-value: 0.0008), as shown in Figure 7.

Discussion

The findings of the present meta-analysis, which included only RCTs involving individuals with CVD or at high risk of CVD, showed that access to polypills led to enhanced adherence to the recommended combination of drugs compared to usual care. The estimated improvement in medication adherence with polypills was reported by all the included RCTs. The present meta-analysis has also found that polypills are also significantly associated with a reduction in cardiovascular events and an increase in HDL. The meta-analysis conducted by Webster et al. found that the use of polypills is significantly associated with improved medication adherence, LDL-C, and systolic blood pressure [18]. Our study showed high heterogeneity in medication adherence. High heterogeneity might be due to inconsistent methods used to evaluate medication adherence. Two studies used the Medication Adherence Questionnaire (MAQ), which is composed of four questions [10, 13], while other included trials used self-reported measures like asking participants about the name and dosages of the drug currently being taken [14-16]. Additionally, variation in population characteristics might also have contributed to high heterogeneity. We conducted a subgroup analysis to assess whether the impact of polypills on medication adherence varies among patients with or without a history of CVD. Medication adherence was significantly higher in patients randomized to the polypill group, irrespective of their CVD histories.

The polypill's accessibility has been shown to increase both patient adherence to and prescription of the recommended combination medication for CVD prevention. This suggests that the positive impacts of the polypill-based strategy are due to changes in both physician behavior (increased levels of the prescription) and patient behavior (greater compliance with prescribed treatments) [6]. Despite evidence of efficacy, a significant gap exists in the implementation of strategies to prevent cardiovascular events in patients with CVD or at high risk of CVD. Barriers include a lack of prescriptions for proven medication therapies for all individuals who may benefit from them. A WHO study in low- and middle-income countries found that only 70.6% of patients with cerebrovascular disease and 81.2% of patients with ischemic heart disease (IHD) were prescribed aspirin, while only 37.8% and 39.8% were prescribed angiotensin-converting enzyme (ACE) inhibitors, respectively [19]. Secondly, poor adherence to prescribed medications is another reason. Non-adherence can have major outcomes because discontinuation is linked with increased rates of mortality and recurrent events in patients with established cardiovascular diseases [20].

Fixed-dose combination drugs (i.e., polypills), especially those based largely on generic preparations, can overcome some but not all of these barriers. Furthermore, when combined with new models of healthcare delivery (e.g., by nonphysician healthcare workers with little or no monitoring), the use of the polypill may lead to more widespread and cost-effective secondary prevention [21].

Our meta-analysis also showed a reduction in cardiovascular events in the polypill group compared to the usual care group. Webster et al. conducted a meta-analysis comparing the efficiency of polypills with usual care in patients with or at high risk of CVD. The previous meta-analysis did not report any significant reduction in cardiovascular events in patients receiving polypills compared to patients in the usual care group [18]. This is possible because of the small number of events overall-only three studies were included in that meta-analysis. The study conducted in 2022 found that the incidence of major cardiovascular events, including death from cardiovascular causes, non-fatal stroke, need for coronary revascularization, and non-fatal myocardial infarction is lower in patients receiving polypills [13].

Regarding the impact of polypills on change in total cholesterol, LDL-C, and HDL from baseline, the present meta-analysis showed that no significant difference was found in change in total cholesterol and LDL-C levels. However, the increase in HDL was significantly higher in patients receiving polypills. Two out of five studies that analyzed the change in LDL-C reported that the reduction of LDL-C was significantly greater in the polypill group compared to the other group [14, 17]. None of the included studies has found any beneficial impact of usual care on LDL-C or total cholesterol.

Based on the data currently available on the individual component medications, the polypill may be widely utilized in secondary prevention and by some high-risk adults without CVD. Prolonged therapy can be expected to reduce risk in these patients by 50% to 75% proportionally [22-23]. The findings of this meta-analysis suggest that the polypill can become an important part of strategies to prevent primary and secondary cardiovascular events in patients with or at high risk of CVD. By improving adherence and simplifying treatment, this method has the ability to reduce the risk of death and cardiovascular events on a global level.

Limitations

The present meta-analysis has certain limitations. Firstly, only six RCTs were included in our meta-analysis. Secondly, we were not able to analyze the primary and secondary prevention of cardiovascular events separately as we did not have access to individual data. One limitation of this meta-analysis is the variability in the methods used to evaluate medication adherence among the included studies. Lastly, outcomes like total cholesterol and HDL were assessed by only three studies. More studies need to be conducted to warrant these findings, including larger sample sizes and the use of a validated medication adherence questionnaire.

## Conclusions

This meta-analysis shows a significant impact of polypills on medication adherence. We also found that polypills can reduce the incidence of cardiovascular events in patients with or at high risk of CVD. Our study has also shown that regardless of the history of CVD, polypills play an important role in promoting medication adherence in patients with and without a history of CVD. The accessibility of a variety of polypills with different ingredients and dosages would increase the variety of treatment options. The research on polypills can be expanded to a larger range of medical diseases, populations, and healthcare systems, as well as outside of high-income nations. In terms of policy, important considerations will be the acceptability of healthcare professionals and patients and the possible effects on healthcare expenditure by individuals and governments.
